# Exogenous acetylsalicylic acid mitigates cold stress in common bean seedlings by enhancing antioxidant defense and photosynthetic efficiency

**DOI:** 10.3389/fpls.2025.1589706

**Published:** 2025-06-13

**Authors:** Barkat Ali, Sujon Kumar, Xiyu Sui, Jianpo Niu, Junqi Yang, Mengni Zheng, Yi Tang, Huanxiu Li

**Affiliations:** ^1^ College of Horticulture, Sichuan Agricultural University, Chengdu, China; ^2^ Triticeae Research Institute, Sichuan Agricultural University, Chengdu, China

**Keywords:** common bean, cold stress, acetylsalicylic acid, physiochemical activity, antioxidant enzymes

## Abstract

Cold stress severely limits the growth and productivity of common bean (*Phaseolus vulgaris* L.) seedlings, particularly during early development. Exogenous application of acetylsalicylic acid (ASA) has proven to be an effective strategy for enhancing cold tolerance. This study investigates the usefulness of exogenous ASA in enhancing cold tolerance in common bean seedlings exposed to cold stress of 5°C for 12 and 24 hours, along with a control (0h). ASA treatments (1 mM and 2 mM) significantly improved critical physiological and biochemical parameters, including photosynthesis, chlorophyll and carotenoid concentrations, oxidative stress markers, malondialdehyde, electrical conductivity, total soluble proteins (MDA, EC, SP), and antioxidant enzyme activity. Under cold stress, ASA2 constantly outperformed the other treatments. Following a 12-hour period, ASA2 showed increased chlorophyll concentrations (8.88%) and augmented *Chl a* levels (21.25%), alongside reducing MDA by 24.96% and SP by 67.1%. After 24 h, ASA2 demonstrated a slight increase in chlorophyll (4.26%) and raised *Chl a* (25.33%), with a significant reduction in MDA (16.5%) and SP (68.3%). ASA1 showed enhancements, mainly in *Chl b* (39.89% at 12 h) and antioxidant enzymes, with notable increases in SOD (113.17% at 12 h) and POD (110.98% at 12 h). Correlation studies indicated significant positive relationships between antioxidant enzyme activity such as, superoxide dismutase, catalase, peroxidase, ascorbate peroxidase (SOD, CAT, POD, and APX) and photosynthetic efficiency. Principal component analysis (PCA) identified ASA2 as the most effective treatment for enhancing stress resilience, accounting for the largest variance in membrane integrity and reduction of oxidative stress. Network analysis further confirmed that ASA2 strengthened the connections between photosynthesis and antioxidant activity, with more resilient and interconnected nodes indicating improved stress adaptability. At 2 mM, ASA upregulated antioxidant genes (*APX1, POD1, SODC*) and photosynthesis genes (*RbcS1, PsbS, POR*), reducing cold-induced oxidative stress and preserving chloroplast function, thereby enhancing cold tolerance and crop resilience under climate stress.

## Introduction

The common bean (Phaseolus vulgaris L.) is a nutritionally rich legume (Bitocchi et al., 2012; Hiz et al., 2014), containing high levels of protein, dietary fiber, complex carbohydrates, essential vitamins, minerals, and phytochemicals with antioxidant properties (Rodriguez Madrera et al., 2021; Yang et al., 2023; Zhang et al., 2023b). Since the mid-20th century, climate change has become a global issue, significantly impacting in the agriculture (Toth et al., 2021). Abiotic stressors increasingly threaten crop yields, potentially reducing global agricultural output (Hirayama and Shinozaki, 2010; Rhaman et al., 2021). Cold stress, in particular, poses severe challenges by hindering plant growth, development, and photosynthesis (Liu et al., 2019).

In common beans, these abiotic stress effects are amplified: photosynthetic efficiency declines 70% compared to maize and tomatoes under cold-stress, while membrane damage is 2–3 times more severe (Sánchez-Reinoso et al., 2018; Mombeni and Abbasi, 2019; Naeem et al., 2020; Iqbal, 2023; Qiao et al., 2024). Common beans are highly vulnerable to low temperatures, with cold injury occurring within 0 to 15°C range (Luo Tong et al., 2005; Miura and Furumoto, 2013; Lv et al., 2023a, Lv et al., 2023a). Symptoms of cold stress include visible signs such as chlorophyll loss, wilting, chlorosis, and necrosis (Bracale and Coraggio, 2003; Saibo et al., 2009). These symptoms transform to catastrophic yield losses after 48 to 72 hours at 10°C, a stress duration that causes only moderate damage in other vegetables (Liu et al., 2019; Vargas et al., 2021; Xue et al., 2023). Prolonged exposure to cold can gradually alter cell structure, reduce photosynthetic efficiency, and increase the production of reactive oxygen species (ROS), exacerbating damage to plant tissues (Allen and Ort, 2001; Thomashow, 2001; Lv et al., 2023a). Plants have developed mechanisms that primarily involve activating antioxidant systems to neutralize the harmful effects of ROS. Enzymes like CAT, POD, SOD, and APX help maintain cellular homeostasis by eliminating ROS and protecting cell membranes from oxidative stress (Miura and Furumoto, 2013; Das and Roychoudhury, 2014).

Recent research increasingly highlights the role of plant growth regulators (PGRs), as salicylic acid (SA) in enhancing plant stress tolerance (Gautam and Singh, 2009; AbdElgawad et al., 2016; Tabassum et al., 2017). These substances, including SA and osmoprotectants, safeguard cell membranes and the photosynthetic system against environmental damage (Foyer and Noctor, 2003). The phenolic compound SA is well-known for its ability to regulate plant growth and development while boosting resistance to both abiotic and biotic stresses (Khan et al., 2003, Tariq Khan et al., 2012). SA enhances plant resilience to environmental stress, facilitating essential processes such as photosynthesis, proline metabolism, and ROS scavenging (Khan et al., 2003; Saleh et al., 2007; Simaei et al., 2012; Miura and Tada, 2014; Ruelland, 2017). Acetylsalicylic acid (ASA), commonly known as aspirin, has a similar molecular structure to SA and has been extensively studied in plant biology for its mechanisms in mitigating stress (Senaratna et al., 2000; Canakci and Munzuroğlu, 2007; Soliman et al., 2018a).

ASA improves plant tolerance to cold stress by upregulating antioxidant enzymes (SOD, CAT, APX) through *NPR1*-dependent and -independent pathways, reducing H_2_O_2_ accumulation by 40% to 60% within 24 h (Daneshmand et al., 2010b; Miura and Tada, 2014; Soliman et al., 2018a). ASA maintains membrane integrity by enhancing phospholipidogenesis and inhibiting lipid peroxidation, as evidenced by a 30% to 50% reduction in Malondialdehyde (MDA) levels compared to untreated plants (Samadi et al., 2019; Li et al., 2022). ASA activates the *CBF/DREB1* cascade, leading to a 2-3-fold increase in proline accumulation and enhanced synthesis of cryoprotectants (Kabiri and Naghizadeh, 2015; Soliman et al., 2018a), while stabilizing photosystem II through *H*SP*70* induction, reducing the decrease in Fv/Fm under cold conditions by 35-45% (Soliman et al., 2018a). In tomato, SA activated PSII protection mechanisms mediated by *EIN3*-like proteins involving *H*SP*21* and ascorbic acid (Zhang et al., 2023a; Sperdouli et al., 2024). The effects of ASA on cold stress resistance are similar to those of other plant growth regulators, such as jasmonic acid (JA) and abscisic acid (ABA). JA and ABA are recognized for their regulation of stress response genes and modulation of antioxidant defense mechanisms (de Ollas and Dodd, 2016; Yoon et al., 2020; Margay et al., 2024). ASA exhibits a unique mechanism of action, especially in its ability to simultaneously regulate photosynthetic efficiency and antioxidant pathways. The roles of JA and ABA in regulating stress-induced gene expression and stomatal control have been extensively studied (de Ollas and Dodd, 2016; Li et al., 2024), while the effects of ASA on photosynthesis under cold stress have received relatively little attention. This study highlights the dual function of ASA: protecting the photosynthetic system and enhancing antioxidant defense mechanisms. Despite these protective effects of ASA, its tissue-specific metabolism in cold-sensitive crops such as common beans is still not well understood and requires further investigation.

Compared to traditional osmoprotectants that require high concentrations (10–100 mM) (Hayat et al., 2012), ASA is effective at relieving stress at minimal concentrations (1–2 mM) due to its stable acetyl group and rapid absorption (Daneshmand et al., 2010a; Kabiri and Naghizadeh, 2015; Soliman et al., 2018a; Matysiak et al., 2020). Empirical evidence suggests that SA increases survival by 20–30% compared to SA alone, which is attributed to its triple mechanism: direct scavenging of ROS (Saleem et al., 2021), membrane stabilization (Moussa and Abdel-Aziz, 2008), and activation of stress genes after hydrolysis to SA (Miura and Tada, 2014). The rapid conversion of ASA can be cleverly combined with cold waves, providing effective protection for high-value legumes, which cannot be met by bulky protectants. Despite its high cost, its low dosage and broad-spectrum efficacy make it a promising cold protection agent in the agricultural field.

Therefore, this study aimed to evaluate the effects of exogenous ASA on improving the tolerance of common bean seedlings to cold stress. This study focused on the effects of ASA on antioxidant enzyme activities, membrane integrity, photosynthetic efficiency, and physiological parameters under cold stress conditions. The aim was to explore whether the application of ASA could alleviate cold stress damage and enhance plant resistance, thereby providing a practical method to ensure crop yield under climate change.

## Materials and methods

### Plant materials

The present study was conducted in a controlled growth chamber at the College of Horticulture, Sichuan Agricultural University, Chengdu, China. The cold-sensitive common bean (*Phaseolus vulgaris* L.) cultivar ‘Bai-Bulao’ was used, obtained from the Department of Horticulture, Olericulture Section. The controlled environment allowed precise regulation of growth conditions to assess the physiological effects of exogenous ASA on common bean seedlings under cold stress.

### Procedure for preparing and sowing seeds

Common bean seeds were soaked in water for 24 hours to facilitate maximum growth. After swelling, the seeds were positioned on Petri dishes lined with damp filter paper to promote germination. Upon reaching a length of roughly 0.5 cm, the white root tips were sown into 32-hole plug trays containing a substrate mixture of peat, vermiculite, and perlite in a 2:1:1 ratio. The trays were subsequently positioned in a growth chamber regulated at 25°C, subjected to a 12-hour light-dark cycle with a light intensity of 30,000 lx. Irrigation was conducted every 48 hours after seedling emergence to promote optimal growth.

### Procedure for preparing and applying ASA

Crystals of ASA with a purity of 99.5% (KESHI Company, China) were used in this experiment. Two concentrations (1 mM, 2 mM) of ASA were prepared along with a control group (ASA0), according to the methods of (Soliman et al., 2018a; Wang et al., 2022). For these concentrations the pH was adjusted to 4.5–5 using 0.05 M NaOH and 0.05 M HCl. The experiment was conducted under three different conditions 0, 12 and 24 hours. Once the seedlings reached the stage of having two leaves and one heart stage, the ASA solutions (1 mM, 2 mM) were applied to the leaves using a hand sprayer. Every seedling was drenched until individual droplets started to run off. The seedlings in the non-ASA control group (ASA0) were treated only with distilled water. Triple replications were conducted for each treatment, including the control, under all three conditions normal growth and cold stress of 12 and 24 hours.

### Treatment with cold stress and sample collection

Twenty-four hours following ASA treatment, the seedlings were exposed to cold stress at a temperature of 5°C for different periods of time (12 and 24 hours). The control groups were sustained at ambient temperature without exposure to cold stress (ASAO). In a completely randomized design (CRD), treatments were organized with three replicates per condition. Biochemical and physiological analysis were conducted on fresh samples obtained from the normal growth seedlings (0h) and at 12 and 24 hours of cold stress. Additional samples were preserved at -80°C for subsequent investigations.

## Measurement of physiochemical parameters

### Photosynthetic indices

Photosynthesis is the process in which plants convert light energy into chemical energy, producing glucose and oxygen from CO_2_ and water. Crucial parameters comprise Pn, Gs, Ci, Tr and chlorophyll content (*Chl*, *Chl a*, *Chl b*), which reflect CO_2_ fixation, gas exchange, and light absorption. During stress, photosynthesis is frequently diminished owing to abridged enzyme activity and pigment injury. Evaluating these parameters supports in measuring stress influences and the efficiency of treatments like ASA in sustaining photosynthetic effectiveness. Photosynthesis (Pn) and associated gas exchange parameters (Pn, Gs, Ci, Tr) were quantified using the (LI-6400XT, Lincoln, NE, USA). Only seedlings exhibiting consistent development and intact leaves were chosen for assessment as given by (Anwar et al., 2018).

### Chlorophyll estimation

Chlorophyll is accountable for light absorption in photosynthesis, which reflects the plants photosynthetic capability. Chlorophyll quantification was performed by preparing a 95% ethanol solution and pouring it into 10 ml tubes. Each tube contained approximately 0.20 g of vein-free leaf samples, which were then stored in the absence of light for around 16 hours until the samples turned bright white. Following that, 300 microliters of each tube’s solution were moved to an enzyme-linked immunosorbent assay (ELISA) plate and looked at with a spectrophotometer at 470, 649, and 665 nm. The following formulas was used to quantify chlorophyll as given by (Arnon, 1949).


$$Chl\;a\;content\;(\frac{mg}{g})=(13.95OD665-6.88OD649)\times V/(W\times 1000)$$

$$Chl\;b\;content\;(\frac{mg}{g})=(24.96OD649-7.320D665)\times \frac{V}{W\times 1000}$$

Cx.c=(10000D470−2.05Ca−114Cb)×V/(245×W×1000)

Whereas *Chl a* and *Chl b* are the concentrations of chlorophyll a and b, respectively and *Cx.c* is the total concentrations of carotenoids

### Assessment of membrane integrity and lipid peroxidation

The effects of cold on membrane integrity and lipid peroxidation were assessed by measuring relative conductivity and MDA levels. Relative conductivity indicates ion leakage and membrane integrity under stress. A leaf disk weighing 0.20 g was immersed in 10 ml of deionized water and the initial electrical conductivity (EC *a*) was measured. After heating the tubes at 100°C for 30 min, the final electrical conductivity (EC *b*) was assessed. Electrolyte leakage (EL) was determined as a percentage using the formula established by: (Jan et al., 2018).


$$Electrolyte\;leakage=\;\frac{\left(EC_{b}-EC_{a}\right)}{EC_{C}}\times 100$$

The variables EC a, EC b, and EC c denote the initial conductivity, conductivity after water bath, and total conductivity, respectively.

MDA is an indicator of lipid peroxidation and membrane damage and is measured to assess oxidative stress. For MDA assay, 1 ml of enzyme extract was mixed with 0.65% thiobarbituric acid (TBA) in 20% trichloroacetic acid (TCA) solution. The solution was heated in a 100°C water bath for 20 minutes, then cooled and centrifuged at 10,000×g for 20 min. Quantify the absorbance at 532 nm, 600 nm, and 450 nm and determine the MDA concentration according to the following formula: (Tiwari et al., 2010).


$$MDA\;µmol/gFW=\frac{\left(C\;\times \;V\;\times \;10-3\right)}{W}$$

C (µmol/L)=6.45  ×(OD532−OD600−0.56 × OD450

Whereas V is the total volume of extract solution (ml); C is the concentration of MDA and W is the fresh weight (g).

### Total soluble proteins

Total soluble proteins (SP) display firmness and metabolic activity during stress. The Coomassie Brilliant Blue G-250 staining technique was employed to quantitatively identify soluble proteins. A volume of 100 µl of the supernatant was combined with 3 ml of Coomassie reagent and allowed to incubate for a duration of 2 min. The absorbance was then measured at a wavelength of 595 nm. The G-250 reagent was synthesized by dissolving 100 mg of G-250 in 50 ml of 90% ethanol, followed by the addition of 100 ml of 85% phosphoric acid. The total volume was then adjusted to 1000 ml. The following formula was used to quantify total soluble proteins as given by (Arora and Wisniewski, 1994).


Y pro (µg)=143.45OD+3.6 (raw) 0r 143.56OD+1.28(park)

$$Protein\;content=(\frac{mg}{gFW})=(3\times Y\times V)/5\times 1000\times a\times W$$

Where Y-reticle value µg; V is total volume of enzyme solution = 3ml; a = 0.1ml; W = 0.2g.

## Antioxidant enzymes estimation

To evaluate the activities of superoxide dismutase (SOD), peroxidase (POD), catalase (CAT), and ascorbate peroxidase (APX), 0.2 g of fresh plant samples were homogenized in 1 ml of ice-cold phosphate buffer (0.05 M, pH 7.8). The mixture was washed with an additional 1 ml of buffer, transferred to a centrifuge tube, and adjusted to a final volume of 5 ml with buffer. The homogenate was centrifuged at 3,000 rpm for 20 min at 0–4°C, and the supernatant was stored at 4°C for future enzyme testing.

SOD activity was measured by evaluating its ability to hinder the photochemical reduction of nitro blue tetrazolium (NBT). A reaction mixture was produced consisting of 0.3 ml 0.75mM *NBT*, 0.3ml 130mM methionine, 0.3ml 0.02mM riboflavin, 0.3ml 0.1mM EDTA-Na2 and 0.25ml distilled water pH 7.8. 30 µl of enzyme extract were added to 3 ml of this mixture. The mixture was placed under fluorescent light at 4,000 lux for 30 min, while control samples were kept in the dark. Absorbance was quantified at 560 nm, and SOD activity was determined using the formula established by: (Beauchamp and Fridovich, 1971).


Total SOD activity(µ/g FW)=[(Ack−AE)]×V]/(0.5Ack×W×Vt)

Whereas Ack is the absorbance of the light control tube; AE is the absorbance of the sample tube; V is the total volume of the sample solution; Vt is the sample dosage (ml) at the time of determination and W is the sample weight at the time of measurement.

POD activity was assessed using guaiacol as a substrate. The reaction mixture consisted of 0.1 M phosphate *buffer* (pH 6.0), 28 μl guaiacol, and 19 μl 30% H_2_O_2_. To 3 ml of this mixture, 100 µl of enzyme extract was added. The absorbance at 470 nm was recorded at 30 s intervals over 3 min, and POD activity was determined using the method established by: (Quintanilla-Guerrero et al., 2008; Muñoz-Muñoz et al., 2009).


Total activity of POD(ΔOD470m−1g−1FW)=(ΔOD×V)/(a×W×t)

Whereas V = 5ml; a = 0.1ml; W = 0.2g and t = 0.5 min

CAT activity was assessed by observing the decomposition of H_2_O_2_ at a wavelength of 240 nm. Prepare a reaction mixture containing 0.1M phosphate buffer (pH 7.0) and 5 ml of 0.1M H_2_O_2_. To 3 ml of this mixture, 100 µl of enzyme extract was added. Absorbance was measured at 0, 1, 2, and 3 min, and catalase activity was determined using the formula established by: (Aebi, 1984).


CAT total activity (ΔOD240min−1g−1FW)=(ΔOD240×V)/(a×W×t)

Whereas V = 5ml; a = 0.1ml and W = 0.2g.

APX activity was assessed by quantifying the oxidation of ascorbic acid at 290 nm. Prepare a reaction mixture using 122 ml of mother liquor A (Na_2_HPO4·12H_2_O), 78 ml of mother liquor B (NaH_2_ PO_4_ ·2H_2_O), 56.8 μl of H_2_O_2_ and 0.0352 g of ascorbic acid. To 3 ml of this mixture, 100 µl of enzyme extract was added. The absorbance at 290 nm was recorded at 30 s intervals over 3 min, and APX activity was determined using the formula established by: (Nakano and Asada, 1981).


APX total activity=[Δa290×VT]/(W×Vs×0.1×t)(µg−1)

ΔA290 is the change of absorbance; W is the fresh weight of the sample (0.2g); t is the reaction time (min); Vt is the total volume of enzyme solution (5ml) and Vs is the volume of the enzyme solution taken during the measurement (0.1ml).

### Expression of gene related to cold stress

#### RNA extraction and RT-qPCR

Total RNA was extracted from the leaves of each treatment group using the RaPure Total RNA Plus Kit (Guangzhou Meiji Biotechnology Co., Ltd., China; http://www.magentec.com.cn), according to the manufacturer’s instructions. *RNA* concentration was measured using a Nano-Drop 2000 spectrophotometer (Thermo Fisher Scientific, Wilmington, DE, USA). First-strand cDNA synthesis was performed using the PrimeScript™ RT Reagent Kit with the gDNA Eraser (TaKaRa, Maebashi, Japan), following the manufacturers protocol. Gene-specific primers were designed using Primer Premier 5. Quantitative reverse transcription polymerase chain reaction (RT-qPCR) was conducted using the iScience SYBR Green I qPCR Mix on a CFX96 Real-Time PCR System (Bio-Rad, Hercules, CA, USA). *β-Actin* was employed as the reference gene for normalization, and relative gene expression levels were calculated using the 2^^−ΔΔCT^ method (Livak and Schmittgen, 2001). Expression differences in response to various ASA treatments were analyzed using R software (version 4.4.0). RT-qPCR was performed with three independent technical replicates and three biological replicates. The specific primer sequences for the 14 target genes are provided in **Supplementary Table S2**.

### Statistical analysis

Statistical analyses of photosynthetic and biochemical parameters were performed using R software (version 4.4.0) for Windows. A two-way analysis of variance (ANOVA) followed by the least significant difference (LSD) test was employed to assess significant differences among treatment means at a 95% confidence level (*P* ≤ 0.05). Results are presented as mean ± standard error (SE) based on three independent biological replicates.

## Results

**Figure 1:**
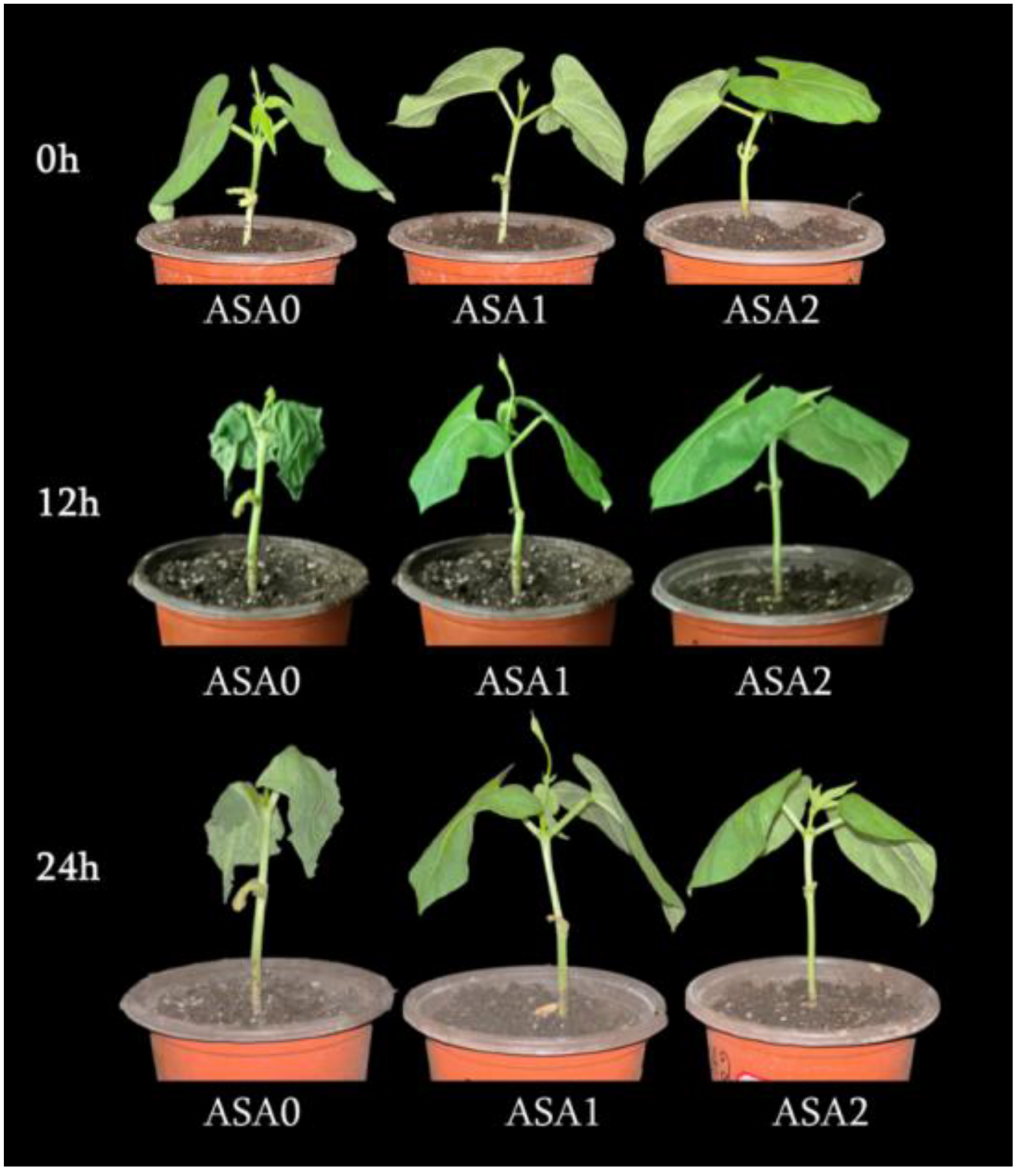
Effect of exogenous ASA on the phenotype of common bean seedlings during 0, 12, and 24 hours of cold stress. Seedlings were treated with 0 mM (ASA0), 1 mM (ASA1), and 2 mM (ASA2) acetylsalicylic acid and then exposed to cold stress (5 °C) for 0, 12, and 24 hours. Phenotypic differences were visibly observed, with ASA-treated plants at 2 mM showing better tolerance, less wilting, and improved leaf turgor compared to the untreated control (ASA0) under cold stress conditions.

Following cold stress, we observed variations in plant phenotypes between the treated and untreated seedlings (**Figure 1a**). The control plants thrived, and ASA facilitated the growth of bean seedlings at the normal temperature. The plants exhibited considerable wilting, possessed pliable petioles, and displayed twisted and desiccated leaves. Seedlings treated with ASA exhibited milder symptoms under cold stress, characterized by minor dehydration of the leaves and slight wilting at the edges.

### Acetylsalicylic acid enhanced photosynthetic parameters

Photosynthesis is an important process in plants because it generates the energy needed for growth, development, and stress response. Under stress conditions such as cold stress, photosynthetic efficiency is often reduced, limiting the plant’s ability to absorb carbon and manage water. Therefore, understanding the effects of treatments like acetylsalicylic acid (ASA) on photosynthesis during stress is critical to enhance plant resilience and efficacy.

**Figure 2:**
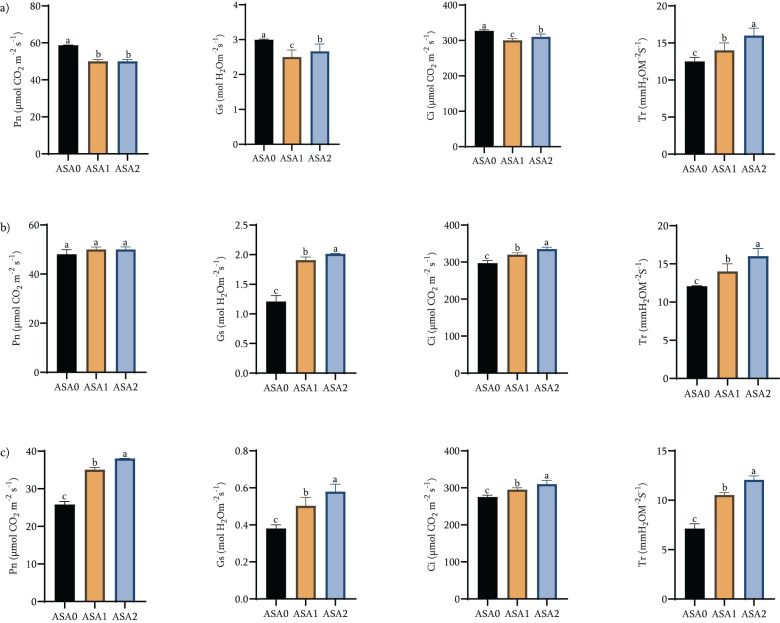
Effects of acetylsalicylic acid (ASA) on net photosynthetic rate (Pn), stomatal conductance (Gs), intercellular CO_2_ concentration (Ci), and transpiration rate (Tr) in common bean under cold stress. Measurements were taken at normal conditions 0h **(a)**, 12 h **(b)**, and 24 h **(c)** of cold stress.

After cold stress, the photosynthetic parameters (Pn, Gs, Ci and Tr) of ASA-treated bean seedlings changed significantly, as shown in **Figure 2**. Under standard growth conditions (**Figure 2a**), photosynthetic parameters changed significantly among ASA0, ASA1 and ASA2. The Pn, Gs, Ci, and Tr values ​​of ASA0 were significantly high compared with ASA1 and ASA2. ASA1 exhibits the lowest values ​​on all parameters, while ASA2 exhibits superior performance compared to ASA1, but is still inferior to ASA0. The results showed that these treatments affected gas exchange and photosynthesis under standard conditions, with the photosynthetic activity of ASA0 (control) exceeding that of the ASA treatment (*p <* 0.05).

During cold stress, ASA treatment showed a significant increase in photosynthetic efficiency. After 12 h of cold stress (**Figure 2b**), Pn (119.05%), Gs (147.89%), Ci (113.14%), and Tr (133.33%) were significantly increased in ASA2 compared with ASA0 (100%), while ASA1 only showed a slight increase of 114.29% (*p <* 0.05). These enhancements became evident after 24 h of cold stress (**Figure 2c**), with significant increases (*p <* 0.05) in Pn (146.90%), Gs (152.63%), Ci (112.66%), and Tr (166.88%) of ASA2 compared to ASA0 (100%). ASA1 showed moderate enhancement in Pn (121.90%), Gs (130.79%), Ci (106.73%), and Tr (124.91%) compared with ASA0 (100%). While ASA1 showed some improvements in photosynthesis metrics, these improvements were significantly less pronounced than ASA2. The results clearly showed that 2 mM ASA was superior to ASA1 and ASA0 in alleviating cold stress and improving gas exchange efficiency (**Supplementary Table S1a**).

### ASA shielded chlorophyll under cold stress

Chlorophyll is the green pigment found in plants and is essential for photosynthesis because it captures light energy and converts it into chemical energy. This process is crucial to the development and sustainability of the plant. Carotenoids are a unique class of pigments that promote light absorption and provide protection against oxidative damage, especially under stressful conditions such as cold stress. Maintaining chlorophyll and carotenoid levels during stress is crucial in maintaining plant health and resilience. This study evaluated the effects of ASA0, ASA1 and ASA2 treatments on chlorophyll and carotenoid levels under standard growth conditions (0 h) and during 12 and 24 h cold stress. Our results demonstrated the impact of these treatments on chlorophyll concentrations and their potential to improve recovery under cold stress.

**Figure 3:**
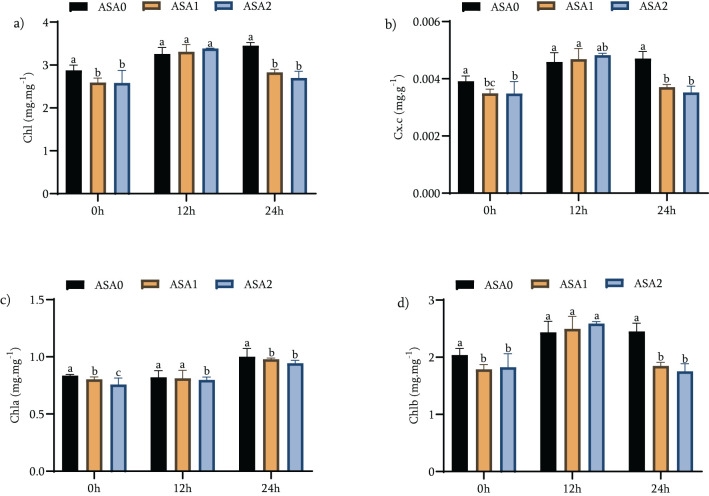
Effects of acetylsalicylic acid (ASA) on **(a)** total chlorophyll (Chl), **(b)** total carotenoid concentration (Cx.c), **(c)** chlorophyll a (Chla), and **(d)** chlorophyll b (Chlb) in common beans under cold stress. Measurements were taken under normal conditions (0 h), 12 h, and 24 h of cold stress.

Under standard growth conditions, both ASA1 and ASA2 treatments significantly increased chlorophyll and carotenoid concentrations compared to the control (ASA0). ASA2 specifically increased total chlorophyll by 20.21% and total carotenoids by 33.33%, while ASA1 increased *Chl b* by 19.70% and *Chl a* by 20.48% as shown in **Figure 3** (*p <* 0.05). These findings suggest that ASA treatment can have a positive effect on pigment production even in the absence of stress. After 12 hours of cold stress, ASA2 showed a significant capacity to maintain chlorophyll content, with a slight increase of 8.88% relative to ASA0. Furthermore, ASA2 preserved *Chl a* content with a significant increase of 21.25% (**Figure 3c**). In contrast, the total chlorophyll content increased significantly by 27.41% (*p <* 0.05) for ASA1 and 39.89% for *Chl b* as shown in **Figure 3a** (*p <* 0.05). These findings highlight the efficacy of ASA1 in rapidly increasing chlorophyll levels during initial cold stress, but ASA2 excels in maintaining pigment stability. After 24 h of cold stress, the effects of ASA1 and ASA2 diverge. ASA1 significantly increased total chlorophyll by 31.01%, indicating a continued beneficial effect on chlorophyll synthesis under long-term cold stress (**Figure 3a**). ASA2 moderately increased total chlorophyll by 4.26% (*p <* 0.05) while effectively maintaining *Chl a* levels by 25.33% (**Figure 3c**). *Chl b* of ASA1 significantly increased by 41.76% as shown in **Figure 3d** (*p <* 0.05), thus enhancing its function of increasing pigment content under long-term stress.

These findings emphasize the specific benefits of ASA1 and ASA2 in increasing chlorophyll concentrations during cold stress. ASA2 was characterized by a superior ability to maintain chlorophyll and carotenoid levels during the first 12 h of cold stress, but ASA1 showed a more pronounced ability to increase chlorophyll content and *Chl b* after 24 h of cold stress. This suggests that ASA1 may be superior at enhancing pigment synthesis over long-term stress, but ASA2 is good at maintaining pigment integrity under stress conditions. Both ASA treatments ultimately enhanced resistance to cold stress by enhancing photosynthetic pigments, indicating their potential use in enhancing plant tolerance to environmental stress.

### ASA mitigated oxidative stress and maintained membrane integrity

Oxidative stress in plants can significantly damage cellular structures, leading to membrane damage, increased ion leakage, and reduced protein levels. Malondialdehyde (MDA) is a major indicator of oxidative stress and represents membrane lipid peroxidation. Electrical conductivity (EC) indicates membrane integrity, while soluble protein (SP) quantifies cellular protein concentration, which is critical for stress response. Maintaining membrane integrity and protein concentration is critical for improving cold stress recovery.

**Figure 4:**
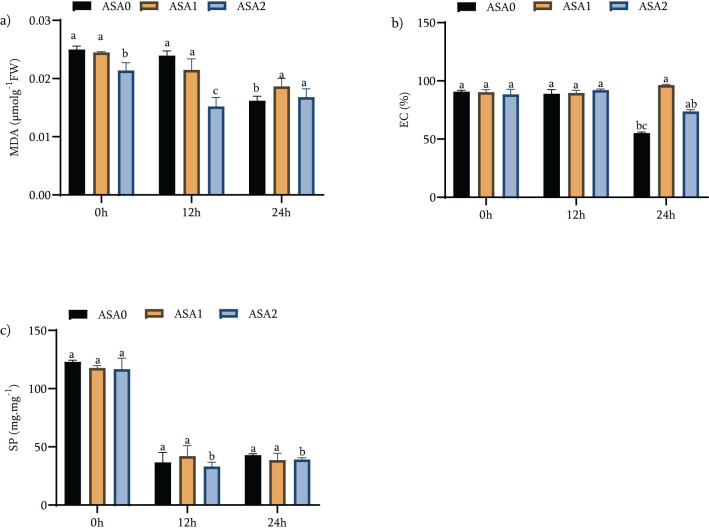
Effects of acetylsalicylic acid (ASA) on **(a)** Malondialdehyde content (MDA), **(b)** electrical conductivity (EC) and **(c)** total soluble protein (SP) in common bean under cold stress. Measurements were taken under normal conditions (0h), 12 h, and 24 h of cold stress.

Under standard growth conditions (0 h), ASA0 had the highest levels of oxidative stress markers: EC, MDA, and SP, all established at 100% of baseline values. ASA1 reduced MDA by 2.02% and SP by 70.2%; however, these reductions were relatively small (**Figure 4**). In contrast, ASA2 showed the greatest reduction (*p <* 0.05) in both MDA (39.23%) and SP (65.1%), indicating a stronger protection against oxidative damage and a more significant increase in protein content. ASA2 showed enhanced efficacy in attenuating oxidative damage after 12 hours of cold stress. MDA levels were reduced by 24.96%, EC by 4.25%, and SP by 67.1% (*p <* 0.05), indicating the ability of ASA2 to maintain membrane integrity and protein levels under stress (**Figure 4**). In contrast, the effect of ASA1 was small, with only a 1.6% reduction in MDA and a 3.14% reduction in SP, neither of which reached statistical significance (*p <* 0.05), indicating that the efficacy of ASA1 during early cold stress is limited. After 24 h of cold stress, ASA2 showed superior performance, reducing MDA by 16.5%, SP by 68.3%, and EC by 16.9% (*p <* 0.05), highlighting its ability to maintain cell function during long-term cold stress (**Figure 4**). In contrast, ASA1 showed the smallest decrease, with only SP showing a statistically significant decrease (*p <* 0.05). These findings highlight the function of ASA2 in maintaining membrane integrity and protecting cellular proteins during prolonged cold stress.

In conclusion, ASA2 showed a significantly enhanced ability to reduce oxidative stress markers (MDA and EC) and maintain SP, particularly under cold stress conditions. Although ASA1 was advantageous, its effect was reduced, exclusively during prolonged cold stress (24 h). The findings indicate that ASA2 is more effective in enhancing cold stress tolerance by significantly reducing oxidative damage and maintaining cell function, making it a possible option for enhancing plant resistance under adverse environmental conditions

### ASA boosted antioxidant enzymes activity

Plants rely on antioxidant enzymes like SOD, POD, CAT, and APX to mitigate oxidative damage during stress. These enzymes are crucial for neutralizing ROS and protecting cellular components. The ability of plants to initiate these responses is critical for survival under adverse climatic conditions such as cold stress.

**Figure 5:**
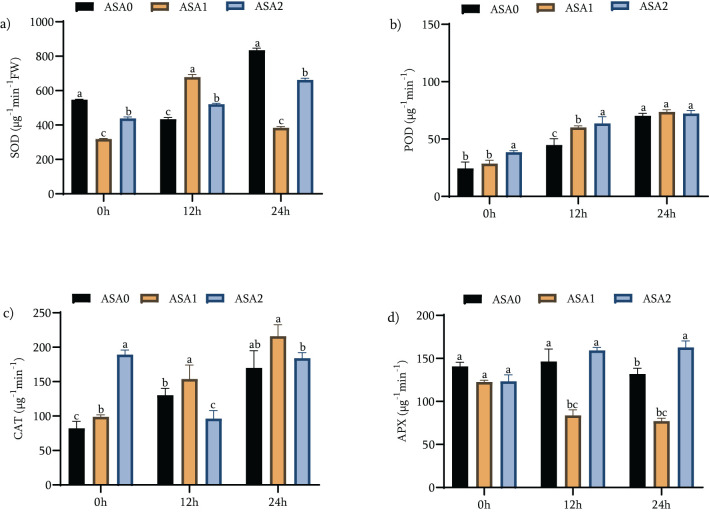
Effects of acetylsalicylic acid (ASA) on **(a)** superoxide dismutase (SOD), **(b)**; peroxidase (POD), **(c)** catalase (CAT), and **(d)** ascorbate peroxidase (APX); in common bean under cold stress. Measurements were taken under normal conditions (0h), 12 h, and 24 h of cold stress.

Under standard growth conditions, ASA2 significantly increased the activities of all evaluated antioxidant enzymes. Relative to the control treatment (ASA0), SOD activity increased by 52.64%, POD by 188.98%, and CAT by 106.7% (**Figures 5 a-c**). The observed results suggest that ASA2 is particularly effective in enhancing antioxidant defense in non-stressful situations. ASA1 showed significant increases, especially in POD (83.98%) and CAT (58.27%). Nonetheless, the APX activity of ASA2 was moderately but significantly reduced (6.29%) compared with ASA0, suggesting that there may be a trade-off in APX activity when other antioxidant enzymes are enhanced. ASA2 showed enhanced antioxidant activity after 12 h of cold stress. SOD increased by 30.96%, POD by 157.77%, and CAT by 118.49% (*p <* 0.05), all of which were significantly higher than the levels recorded in ASA1. ASA1 showed a more significant increase in SOD (113.17%) and POD (110.98%), although the increase in CAT (55.74%) was smaller than that of ASA2. Both treatments showed significant decreases in APX activity, with ASA1 decreasing by 31.73% and ASA2 decreasing by 37.03% (**Table S1b**), consistent with oxidative stress responses in cold environments. ASA2 significantly enhanced antioxidant enzyme activity after 24 h of exposure to cold. SOD increased by 51.06%, POD by 87.49%, and CAT by 7.8% (*p <* 0.05), indicating its continued ability to enhance oxidative stress defense throughout the stress process. In contrast, ASA1 showed a small enhancement in SOD (18.84%) and POD (65.3%), along with a significant decrease in CAT activity of 49.05% (*p <* 0.05). Notably, APX activity was significantly enhanced by ASA2 by 31.88%, offsetting the decrease noted previously for both treatments during the initial cold shock (**Figure 5d**).

The results showed that ASA2 was significantly better than ASA1 in enhancing antioxidant enzyme activity, particularly during long-term cold stress. The increase in SOD, POD, and CAT activities under normal and cold stress environments indicates that ASA2 can effectively stimulate antioxidant defense. APX activity was significantly enhanced (31.88%) after 24 h of cold stress, emphasizing the ability of ASA2 to enhance overall oxidative stress resistance. Although ASA1 can enhance antioxidant defense, it is not as effective as ASA2, especially under continuing cold stress. The atypical trend in the data specifically, the first decrease in APX activity followed by the resurgence of ASA2–24 h later suggests a complex regulatory process that may require modulation of many antioxidant pathways under stress conditions. ASA2 is a more effective treatment for alleviating oxidative stress and promoting cold stress tolerance, providing a feasible strategy to improve plant resilience

### Correlation between antioxidant enzymes and photosynthesis

Correlation matrix demonstrates the effect of ASA treatment on the balance between photosynthetic efficiency and oxidative stress defense.

**Figure 6:**
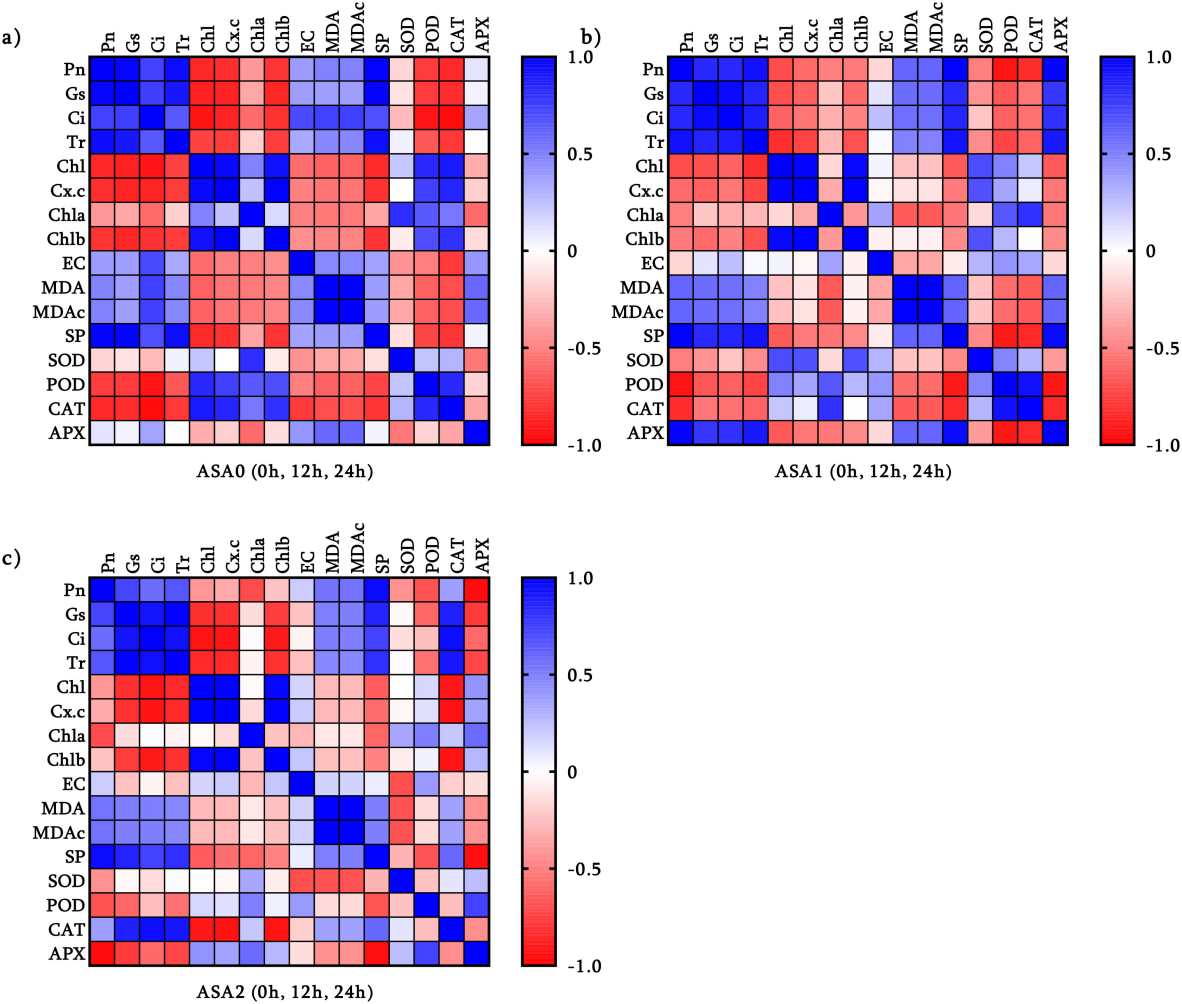
Pearson’s correlation coefficient between three different acetylsalicylic acid (ASA) treatments in common bean under cold stress: **(a)** ASA0, **(b)** ASA1, and **(c)** ASA2. Correlations were analyzed under normal conditions (0h); and after 12 h and 24 h of cold stress.

In the control (**Figure 6a**), photosynthetic parameters (Pn, Gs, Tr) were positively correlated with photosynthetic pigments (*Chl, Chl a, Chl b*), emphasizing their importance in photosynthesis. Nonetheless, antioxidant enzymes (SOD, POD) were negatively correlated with these parameters, indicating that oxidative stress impedes photosynthesis. MDA and SP were negatively correlated, highlighting the deleterious effects of oxidative stress. ASA1 increases photosynthetic efficiency, as evidenced by the strong association with soluble protein (SP) and photosynthetic parameters (**Figure 6b**). Antioxidant enzymes (SOD, CAT*, and* APX) are positively correlated with pigments, indicating enhanced oxidative defense capabilities. Nonetheless, oxidative stress still exists because MDA and EC adversely affect photosynthesis. ASA2 showed the most substantial association. Antioxidant enzymes (SOD, POD, CAT, APX) and photosynthetic parameters (Pn*, Chl, Chl a, Chl b*) showed significant positive correlations, indicating that ASA2 has an excellent ability to enhance photosynthesis and mitigate oxidative damage (**Figure 6c**). The negative correlation between MDA and essential photosynthetic parameters suggests that ASA2 can effectively alleviate oxidative stress.

ASA2 is the most effective treatment for enhancing photosynthesis and antioxidant defense while reducing oxidative stress. ASA1 had certain advantages but could not completely alleviate oxidative damage, while ASA0 showed obvious signs of stress.

### Principal component analysis

**Figure 7:**
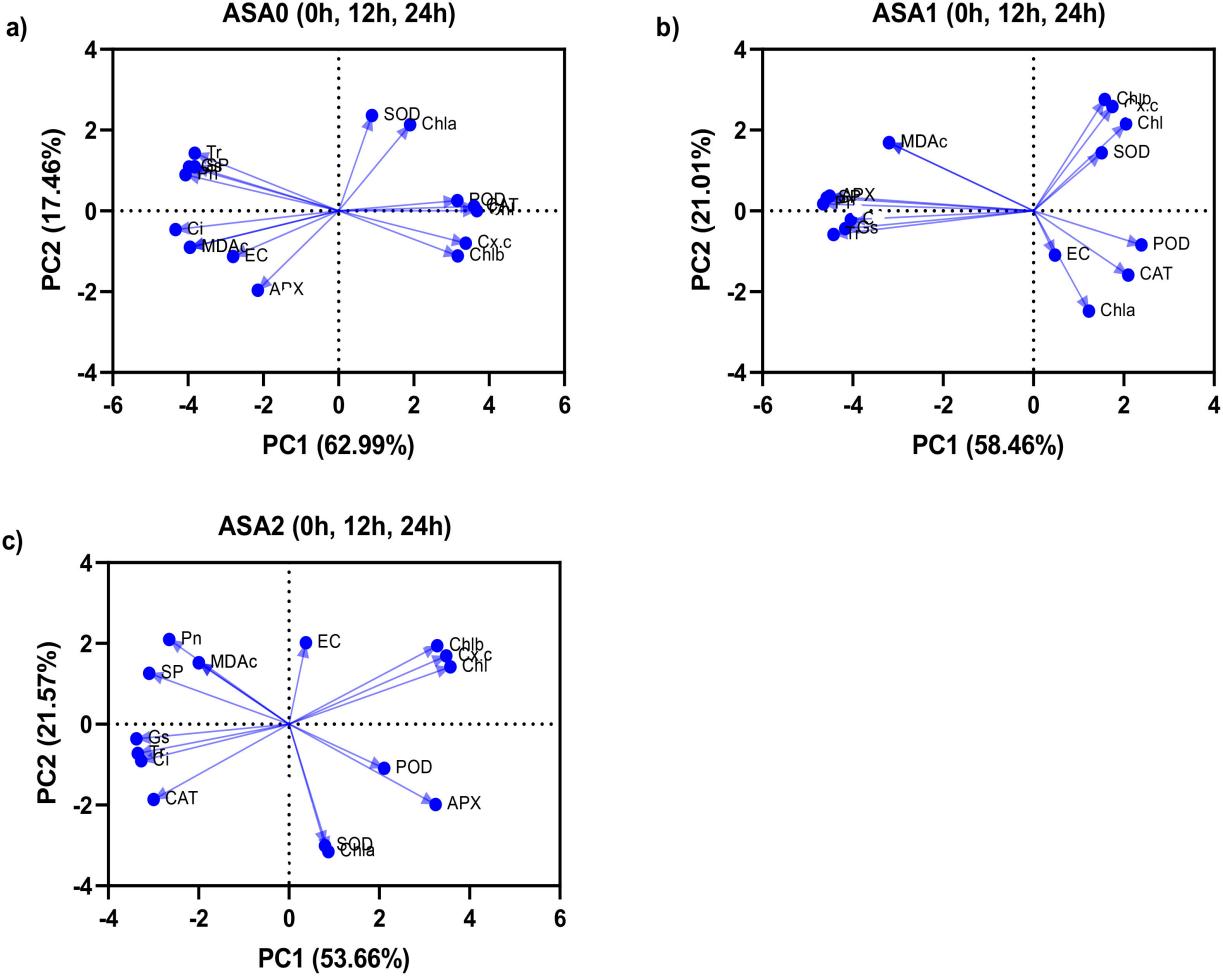
Principal component analysis (PCA) between three different acetylsalicylic acid (ASA) treatments in common bean under cold stress: **(a)** ASA0, **(b)** ASA1, and **(c)** ASA2. Correlations were analyzed under normal conditions (0 h); and after 12 h and 24 h of cold stress.

Principal component analysis was performed between all physiochemical parameters for ASA0 (without ASA) and ASA (ASA1 Mm and ASA2 Mm) treated seedlings under both normal growth (0h) and cold stress of 12 and 24 hours (**Figure 7**). In the control treatment, PC1 accounted for 62.99% of the variance, indicating a strong negative correlation between MDA and EC, indicating membrane instability and oxidative damage (**Figure 7a**). Photosynthetic factors (Pn, Tr*, Chl*) had a favorable effect on PC1; however, their effects were attenuated under stress, indicating impaired photosynthetic performance in the absence of ASA. PC2 accounted for 17.46% of the variance and was related to antioxidant enzymes (SOD, CAT, POD), but its effect was rather small. Oxidative stress had a more pronounced effect on control plants, resulting in significant deterioration in physiological performance. In ASA1 (**Figure 7b**), PC1 accounted for 58.46% of the variance, showing a strong positive correlation with antioxidant enzymes (SOD, CAT, POD, APX) and photosynthetic parameters (Pn*, Chl, Chl a*). This indicates that ASA1 significantly improves photosynthetic efficiency and antioxidant defense. Negative correlations with MDA and EC indicate reduced oxidative stress. PC2 accounted for 21.01% of the variance, focusing on soluble proteins and gas exchange, thus emphasizing the importance of protein stability and gas exchange in the stress response. ASA1 enhances plant recovery by alleviating oxidative stress and enhancing physiological functions. In ASA2 (**Figure 7c**), PC1 accounted for 53.66% of the variation and showed strong connections with antioxidant enzymes (SOD, CAT, POD, APX) and photosynthetic parameters (Pn*, Chl, Chl a, Chl b*). This suggests that ASA2 optimizes the enhancement of photosynthesis and oxidative defense. MDA and EC were negatively correlated with PC1, indicating the absence of oxidative damage. PC2 represented 21.57% of the variance and showed changes in Gs, Ci, and SP, emphasizing the importance of protein stability and stomatal function in stress responses. ASA2 significantly reduces oxidative damage while improving overall plant performance.

PCA results indicated that ASA2 provided the fairest enhancement, improving photosynthesis and antioxidant defense while reducing oxidative stress. ASA1 also improves photosynthesis and antioxidant activity, but the effect is less pronounced than ASA2. The control (ASA0) clearly shows that in the absence of treatment, oxidative stress significantly impairs physiological function.

### Network analysis

**Figure 8:**
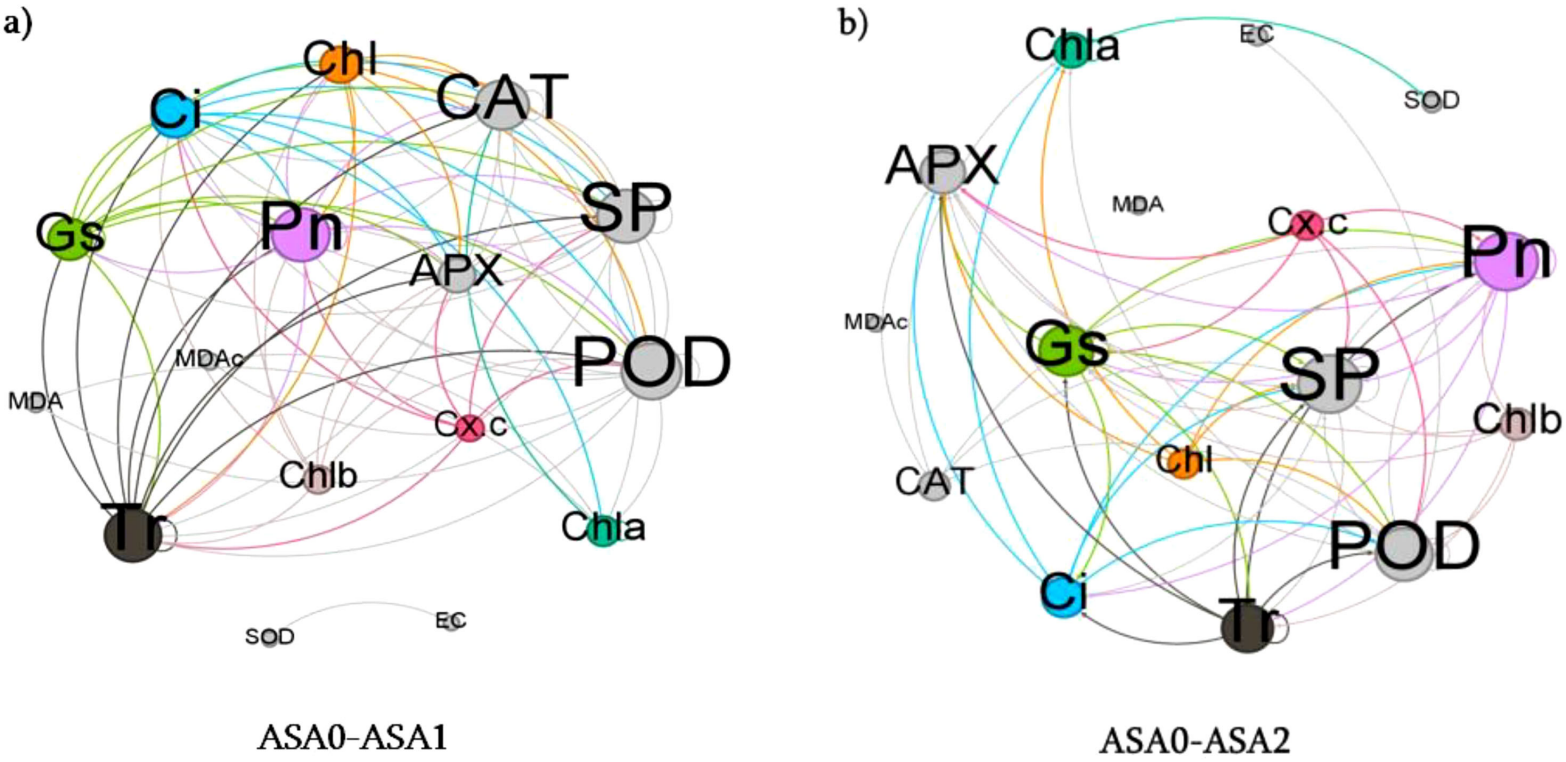
Network analysis of physicochemical parameters across different ASA treatments. The network illustrates the degree of interaction between **(a)** ASA0-ASA1 and **(b)** ASA0-ASA2. The size of each node corresponds to the strength of interactions. The green color represents the strength of positive and red color represent negative interactions.

Network analysis comparing ASA0 and ASA1 showed a more robust positive correlation between antioxidant enzymes (SOD, CAT, APX) and photosynthetic parameters (Pn*, Chl*, SP) in ASA1, as evidenced by the larger green nodes (**Figure 8a**) This suggests that ASA1 can enhance photosynthetic efficiency and antioxidant defense. The reduction in the number and size of red nodes (representing MDA and EC) in ASA1 implies reduced oxidative stress and reduced membrane instability relative to the control (ASA0). ASA1 treatment improved plant physiological performance by enhancing antioxidant activity and photosynthetic activity, as evidenced by enhanced green node connectivity. In the comparison of ASA0 and ASA2, ASA2 exhibits a significantly stronger network. Prominent green nodes representing antioxidant enzymes (SOD, CAT) and photosynthetic parameters (Pn*, Chl*) exhibit tight interconnections, indicating that ASA2 provides enhanced protection by improving antioxidant defense and photosynthetic efficiency (**Figure 8b**). The reduction in the number of MDA and EC red nodes and their limited connectivity suggests that the effects of oxidative stress are greatly attenuated in ASA2-treated plants compared to ASA0. The strong correlation between antioxidant enzymes and photosynthetic indicators further confirms that ASA2 is highly effective in mitigating oxidative stress and improving overall plant performance.

Network analysis showed that ASA2 most broadly enhances antioxidant defense and photosynthetic efficiency while significantly reducing oxidative stress. ASA1 enhances plant stress resistance; however, its effect is less dramatic than ASA2, as evidenced by the reduction in the number and size of green nodes. In the absence of treatment, ASA0 exhibits elevated oxidative stress and decreased physiological performance.

### ASA upregulates antioxidant and photosynthetic genes in cold-stressed common bean

**Figure 9:**
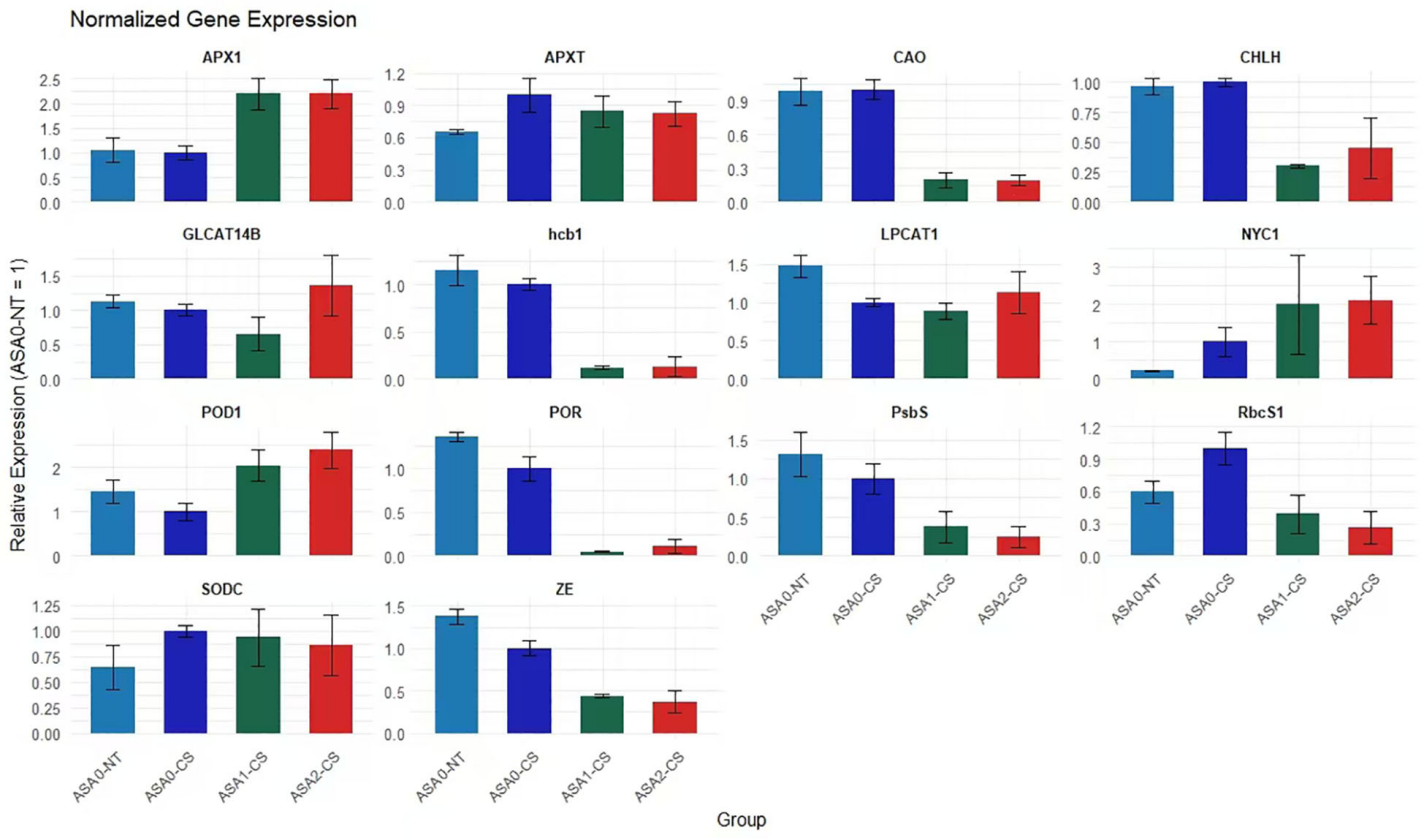
RT-qPCR analysis of gene expression in response to ASA treatment and cold stress. Bar plots show the relative expression levels of 14 selected genes involved in photosynthesis, chlorophyll metabolism, antioxidant defense, and stress response under four conditions: ASA0-CS (cold stress without ASA), ASA0-NT (normal temperature without ASA), ASA1-CS (low concentration of ASA under cold stress), and ASA2-CS (higher concentration of ASA under cold stress). Expression levels were normalized, and error bars represent standard deviation across replicates. Genes *RbcS1*, APX*1*, POD*1*, and *NYC1* show significant upregulation under ASA treatment compared to cold stress alone, while *hcb1, CHLH, and ZE* show downregulation, particularly under ASA2-CS. These results support the role of ASA in modulating gene expression to enhance cold stress tolerance in plants.

Cold stress significantly reduced the expression of genes related to antioxidant defense, chlorophyll, and photosynthesis in common bean seedlings. Though, exogenous ASA alleviated these possessions in a concentration-dependent manner. Fold change analysis showed that antioxidant-related genes, including *APX1*, *POD1*, and *SODC*, were significantly upregulated under ASA treatment, *APX1* expression increased more than 2-fold and nearly 3-fold under ASA1-CS and ASA2-CS treatments, respectively, compared with cold-stressed control seedlings (ASA0-CS), indicating enhanced ROS scavenging ability. Similarly, ASA significantly alleviated the cold-stress-induced repression of photosynthesis genes *RbcS1*, *PsbS*, and *POR*, with the expression of these genes under ASA2-CS treatment increasing more than 2- to 4-fold compared with that under ASA0-CS treatment. In addition, the expression of chlorophyll metabolism-related genes, including *CHLH*, *CAO*, and *NYC1*, increased 2- to 3-fold after ASA treatment compared with the cold stress control. These results indicated that ASA, especially at 2 mM concentration, significantly enhanced the expression of stress-responsive genes, thereby enhancing antioxidant defense and maintaining photosynthetic efficiency under cold stress conditions (**Figure 9**).

**Figure 10:**
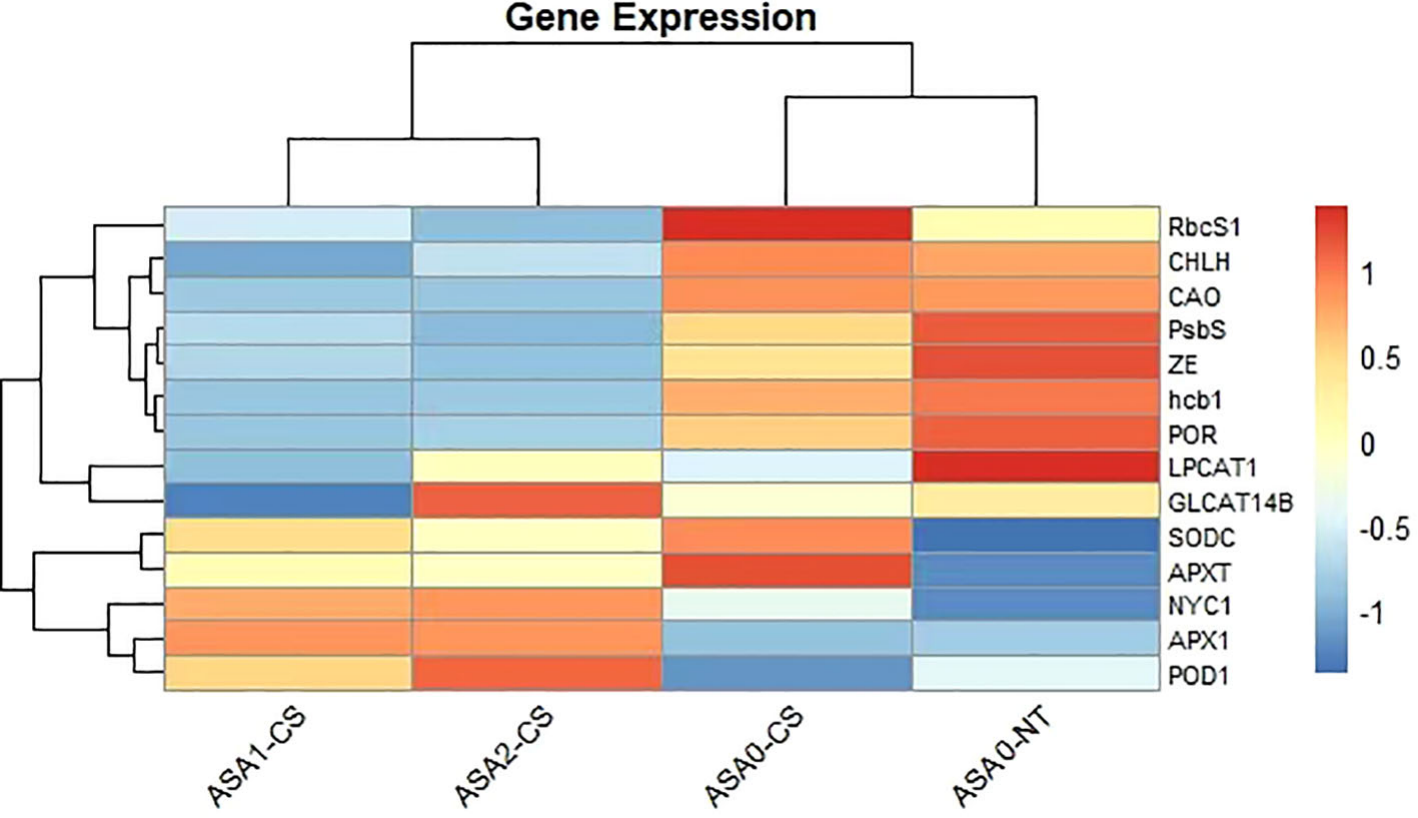
Heatmap of key gene expression profiles in response to ASA treatment and cold stress. The heatmap displays the relative expression levels of selected stress-responsive genes across four treatment groups: ASA1-CS, ASA2-CS, ASA0-CS (ASA-treated under cold stress), and ASA0-NT (ASA-treated under normal temperature). Expression values were standardized and clustered using hierarchical clustering. Red indicates higher expression, blue indicates lower expression, and white represents intermediate levels. Genes *RbcS1, CHLH, PsbS, GL*CAT*14B*, and APX*1* show differential expression patterns in response to ASA treatment and cold stress. Data were obtained through RT-qPCR analysis, and values represent normalized expression changes.

Heatmap depicts differential expression of essential genes related to photosynthesis, antioxidant defense, and chlorophyll metabolism in four treatments (ASA1-CS, ASA2-CS, ASA0-CS, and ASA0-NT). Warm tones (red) indicate upregulation, while cool tones (blue) indicate downregulation. Genes such as *RbcS1*, *CHLH*, and *PsbS* were significantly upregulated under ASA0-CS and ASA0-NT treatments, indicating enhanced photosynthesis activity under untreated and control conditions. In contrast, antioxidant-related genes such as *SODC*, *APXT*, and *POD1* showed enhanced expression under ASA1-CS and ASA2-CS treatments, indicating that ASA treatment enhanced antioxidant defense mechanisms under cold stress. The expression patterns suggest that ASA, especially at 1 mM and 2 mM concentrations, is able to regulate gene networks that mitigate the adverse effects of cold stress on common bean seedlings (**Figure 10**).

## Discussion

**Figure 11:**
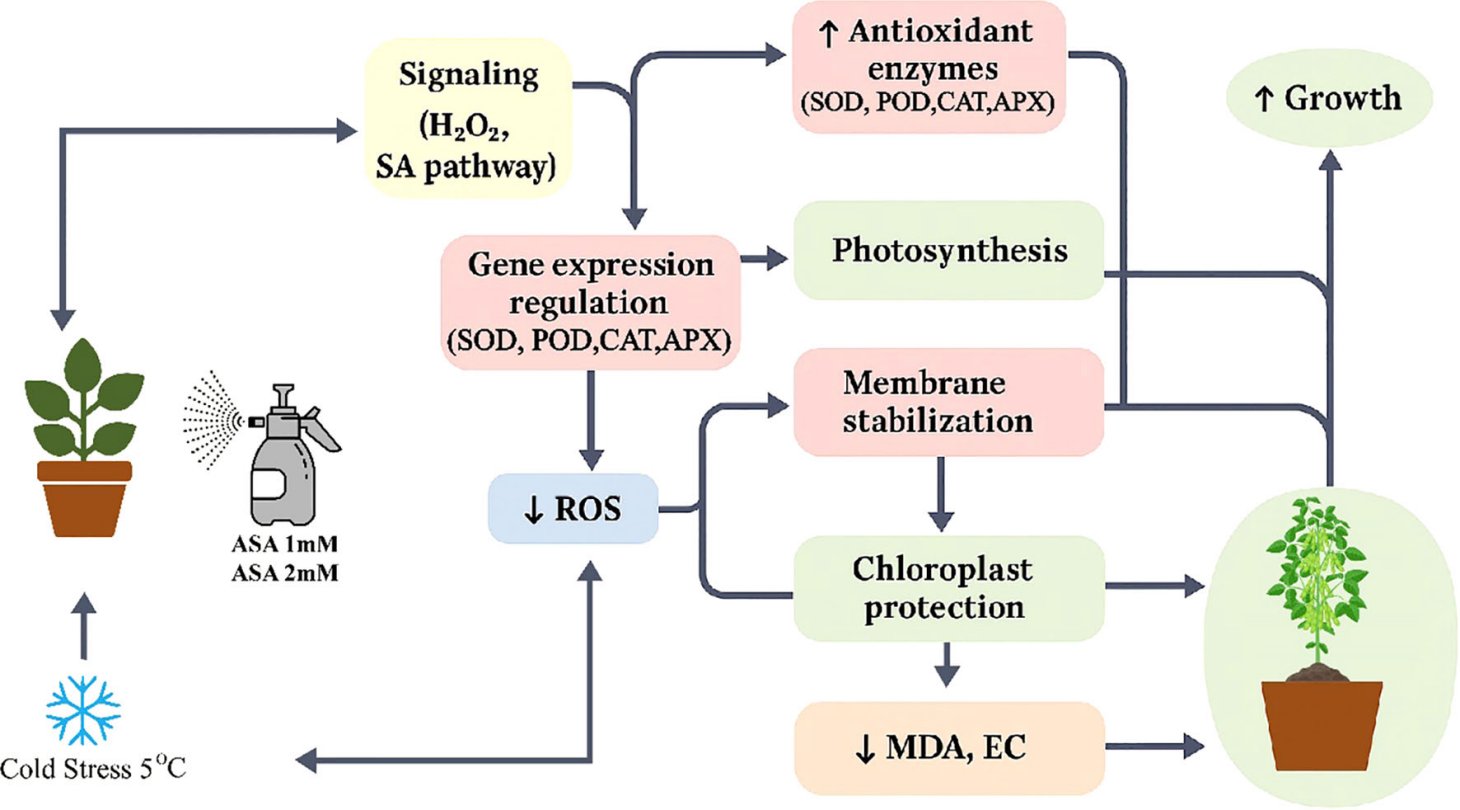
Schematic representation of ASA-induced enhancement of cold stress tolerance in plants. Exogenous application of acetylsalicylic acid (ASA) improves plant growth under cold stress conditions (5°C). ASA treatment enhances physiological and biochemical processes, including: (1) photosynthesis parameters (Pn: net photosynthetic rate, Gs: stomatal conductance, Ci: intercellular CO_2_ concentration, Tr: transpiration rate), (2) chlorophyll content (Chl, Cx·c; Chla, Chlb); (3) antioxidant enzyme activities (SOD, superoxide dismutase; POD, peroxidase; CAT, catalase; APXm ascorbate peroxidase), and (4) biochemical indicators such as soluble protein (SP). Meanwhile, cold-induced damage indicators like Malondialdehyde (MDA) and electrolyte conductivity (EC) are reduced. These responses contribute to improved cold stress tolerance and plant growth.

In this study exogenous acetylsalicylic acid (ASA) significantly improved the cold stress tolerance of common bean seedlings, indicating that it has the potential to be an effective method to alleviate abiotic stress. Cold stress significantly reduces the photosynthetic efficiency, enzymatic activity, and membrane stability of plants (Banerjee and Roychoudhury, 2019; Ali et al., 2022), and ASA has been shown to have the potential to alleviate these adverse effects (**Figure 11**).

### ASA and photosynthesis

Cold stress usually reduces photosynthetic rate by limiting stomatal conductance and impairing chlorophyll stability (Fu et al., 2016; Bhattacharya, 2022). This study showed that application of 2 mM concentration of ASA (ASA2) could maintain higher photosynthetic parameters (Pn, Gs, Ci, Tr) under cold stress, indicating that ASA has a protective effect on photosynthesis. ASA2 can maintain chlorophyll (*Chl, Chl a, Chl b*) and carotenoids (*Cx.c*), which are essential for light absorption and energy conversion. This is consistent with the findings that SA can stabilize the photosynthetic apparatus, thereby improving light energy utilization (Moustakas et al., 2023; Sperdouli et al., 2024). Similarly, ASA2 maintained chlorophyll content, increasing *chl a* by 21.25% and *chl* content by 8.88%. This finding was also confirmed by the study demonstrated that SA treatment can alleviate oxidative damage and photoinhibition under stress conditions (Yang et al., 2019). At lower concentrations (ASA1, 1 mM), ASA increased the levels of photosynthetic pigments, indicating that low doses can also be effective, as pointed out by (Daneshmand et al., 2010b). Similarly, ascorbic acid treatment in wheat enhanced the responses of photosynthetic pigments, osmoprotectants, and antioxidant enzymes to salt stress (Siddiqui et al., 2018). Correspondingly, several plant growth regulators, including jasmonic acid (JA) and abscisic acid (ABA), have been documented to increase chlorophyll content and photosynthetic efficiency under stress conditions (Awan et al., 2021; Tariq et al., 2022).

### ASA and oxidative stress mitigation

Cold stress leads to the formation of ROS, which cause oxidative damage to cell membranes, proteins, and lipids (Dreyer and Dietz, 2018; Juan et al., 2021; Manasa S et al., 2022). MDA, an indicator of lipid peroxidation, and EC decreased in seedlings treated with ASA under cold stress (MDA decreased by 39.23% after 12 h and by 24.96% after 24 h), confirming the protective role of ASA in maintaining membrane integrity. This is consistent with earlier studies, showed that SA and other plant growth regulators (*PGRs*) mitigate oxidative damage by enhancing membrane stability (Paul et al., 2024; Kaya et al., 2023). Similarly, Studies have shown that exogenous SA can alleviate salt stress in mustard plants by enhancing their growth, physiological and biochemical parameters, and antioxidant enzyme activities (Islam et al., 2023). The reduction in EC further confirmed the improved membrane stability, as increased EC levels are associated with cell damage and membrane permeability (Sun et al., 2020; Gurova and Denisyuk, 2021).

### ASA and antioxidant enzyme activities

Regulating the activity of antioxidant enzymes is the key mechanism by which ASA reduces oxidative damage. In the current study ASA1 (1 mM) significantly increased SOD activity by 113.17% after 12 hours of cold stress, while ASA2 increased the activity of POD and CAT by 157.77% and 118.49% respectively after 24 hours. The increase in the activity of SOD, CAT and POD is associated with the enhancement of ROS scavenging ability and the reduction of oxidative damage (Gill and Tuteja, 2010; Azarabadi et al., 2017; Fujita and Hasanuzzaman, 2022). The increase in CAT and POD activities may indicate an alteration in the antioxidant pathways, thereby enhancing the detoxification of ROS under cold stress (Valizadeh-Kamran et al., 2018). Similar changes in antioxidant processes have been observed in other studies on *PGR* treatments, in which SA and *JA* enhance stress responses by reconfiguring antioxidant pathways (Khan et al., 2020; Ghassemi-Golezani and Farhangi-Abriz, 2021; Sabagh et al., 2021; Samanta and Roychoudhury, 2025). Although SA has been documented to affect antioxidant enzyme activities, other plant growth regulators, like *JA* and *ABA*, have also been shown to affect the response of the antioxidant system to abiotic stress (Saxena et al., 2019; Tariq et al., 2022).

### ASA-mediated antioxidant–photosynthesis crosstalk

Principal component and network studies showed that ASA treatment, especially ASA2, promoted a synchronized response between antioxidant defense and photosynthetic efficiency under cold stress. In both ASA1 and ASA2 treatments, there were significant positive correlations between antioxidant enzymes (SOD, CAT, APX) and photosynthetic parameters (Pn*, Chl*, SP), suggesting a mechanistic interaction: enhanced ROS scavenging capacity (SOD) could protect the structure and function of chloroplasts, thereby maintaining CO_2_ uptake and chlorophyll integrity The interaction between antioxidants and photosynthesis was reflected in the reduction of oxidative stress indicators (MDA, EC) and the enhancement of physiological performance in ASA-treated seedlings. The SOD*-*Pn interaction showed that superoxide dismutation is essential for maintaining electron transport efficiency and reducing photooxidative damage. ASA2 showed the most resilient integrated network, indicating its enhanced ability to maintain membrane stability and photosynthetic function under cold stress. These results confirm previous studies that SA derivatives enhance plant stress resilience by enhancing antioxidant capacity and optimizing photosynthetic efficiency (Farouk et al., 2020; Yang et al., 2023).

During cold stress, APX activity unveiled a biphasic response to ASA treatments. In seedlings treated with ASA1 and ASA2, APX activity decreased significantly after 12 h, with a more decrease at the 2 mM ASA treatment. This early inhibition may indicate a transient alteration in antioxidant defense mechanisms, in which ASA affects the redox environment and redistributes ROS scavenging functions between various enzymes (Caverzan et al., 2012; Rajput et al., 2021). Consistent with recent studies, APX is markedly sensitive to the cellular redox environment and may be transiently inhibited when other pathways, such as SOD or POD, are temporarily favored to control acute oxidative surges (Gill and Tuteja, 2010; Sharma et al., 2012). Remarkably, APX activity was significantly enhanced under ASA2 treatment at 24 h of cold stress, indicating that its activation was time-dependent. This pattern suggests that ASA may first inhibit APX activity as a rapid defense response but then reactivate it to maintain a sustained antioxidant balance during plant adaptation to prolonged cold stress (Duan et al., 2012). This suggests that ASA2 can provide a more durable and stable antioxidant defense under long-term cold stress, which is consistent with previous research results (Wu et al., 2024). SA analogs such as ASA are recognized for their ability to modulate gene expression and enzyme activity through redox-sensitive signaling pathways and hormone interactions (Mittler, 2002; Koornneef et al., 2008; Caarls et al., 2015; Khan et al., 2015), which may play a role in the subsequent enhancement of APX. The temporal regulation of APX activity during ASA treatment suggests the existence of a dynamic and coordinated antioxidant response, which improves the cold stress tolerance of common bean seedlings.

### Gene expression modulation by ASA

At the molecular level, ASA regulates the expression of essential genes related to photosynthesis and antioxidant defense. Cold stress (ASA0-CS) reduced the expression of multiple genes related to ROS detoxification, such as APX*1*, POD*1*, and SOD*C*, leading to increased ROS levels and oxidative damage (Karami-Moalem et al., 2018; Gorpenchenko et al., 2023). However, ASA treatment, especially 2 mM concentration of ASA (ASA2-CS), counteracted these effects by upregulating the expression of antioxidant genes and enhancing ROS scavenging ability. Similar results were obtained with the exogenous application of abscisic acid (*ABA*) which enhanced antioxidant enzyme activities and related gene expressions, particularly SOD and POD, thereby improving plant tolerance to cold-induced oxidative stress (Guo et al., 2012). SA analogs and ASA can enhance the expression of antioxidant genes in response to cold stress (Akçay et al., 2024; Soliman et al., 2018a). In addition to antioxidant genes, ASA also restored the expression of genes related to photosynthesis (*RbcS1, PsbS, and POR*), emphasizing its function in protecting the photosynthetic apparatus under stress. Studies have shown that SA mitigates aluminum toxicity in alfalfa by maintaining chloroplast integrity and enhancing the expression of photosynthetic genes (Cheng et al., 2020). Similarly exogenous hemin can alleviate Cd stress in maize by promoting leaf photosynthesis (Piao et al., 2022). These findings are consistent with studies in other legumes, including mung bean and broad bean, in which *PGRs* enhanced the expression of genes related to stress tolerance (Tuteja et al., 2010; Reddy et al., 2012; Divi et al., 2016; Büyük and Aras, 2017).

## Conclusion

In summary, the utilization of ASA, particularly at a dose of 2 mM (ASA2), presents considerable promise for enhancing cold stress resilience in common bean seedlings. ASA not only maintained photosynthetic efficiency and chlorophyll integrity but also reduced oxidative damage by regulating antioxidant enzyme activity and gene expression. These results align with prior research on SA and other PGRs, indicating that ASA may serve as an effective means to bolster plant resilience against abiotic stressors. Subsequent investigations should concentrate on the intricate molecular processes through which ASA influences gene expression and enzymatic activity to enhance its utilization in agricultural contexts. Furthermore, comparing the effects of ASA with those of other plant growth regulators such as *JA* and *ABA* may yield additional insights into their synergistic roles in stress tolerance.

## Data Availability

The original contributions presented in the study are included in the article/**Supplementary Material**. Further inquiries can be directed to the corresponding author.
