# Supplementary material for: Exogenous acetylsalicylic acid mitigates cold stress in common bean seedlings by enhancing antioxidant defense and photosynthetic efficiency
Source: Front Plant Sci. 2025 Jun 13;16:1589706. doi: 10.3389/fpls.2025.1589706 (PMC12202537; doi:10.3389/fpls.2025.1589706)
Supplement: Supplementary file 1 [file Table1.docx]

**TABLE S1a |** Effect of acetylsalicylic acid (ASA) on photosynthesis and chlorophyll levels in common bean under cold stress

| Treatment^1^ | Dose^2^ | Pn | Gs | Ci | Tr | Chl | Cx.c | Chla | Chlb |
| --- | --- | --- | --- | --- | --- | --- | --- | --- | --- |
|  |  | (*µmol CO_2_ m^-2^ s^-1^*) | (*mol H_2_O m^-2^ s^-1^*) | (*µmol CO_2_ m^-2^ s^-1^*) | (*mol H_2_O m^-2^ s^-1^*) | (*gm. g^-1^*) | (*gm. g^-1^*) | (*gm. g^-1^*) | (*gm. g^-1^*) |
| 0h | ASA0 | 58.71±3.06a | 2.99±0.26a | 327.18±5.33a | 16.89±2.43c | 2.87±0.07a | 0.003±0.00a | 0.83±0.00a | 2.03±0.06a |
|  | ASA1 | 50.00±0.53b | 2.50±0.00c | 300.00±3.79c | 14.00±0.00b | 3.25±0.08b | 0.004±0.00b | 0.82±0.03b | 2.43±0.11b |
|  | ASA2 | 52.00±0.55b | 2.60±0.06b | 310.00±1.23b | 15.00±1.39a | 3.45±0.04b | 0.004±0.00b | 1.00±0.04c | 2.45±0.08b |
| 12h | ASA0 | 42.00±1.49a | 1.42±0.36b | 296.11±4.63c | 12.00±0.97c | 2.59±0.05a | 0.003±0.00a | 0.8±0.01a | 1.78±0.04a |
|  | ASA1 | 48.00±0.14a | 1.90±0.00a | 320.00±2.93b | 14.00±0.00b | 3.3±0.09a | 0.004±0.00a | 0.81±0.04a | 2.49±0.12a |
|  | ASA2 | 50.00±0.28a | 2.10±0.20a | 335.00±6.02a | 16.00±2.60a | 2.82±0.04a | 0.003±0.00ab | 0.97±0.00b | 1.84±0.03b |
| 24h | ASA0 | 25.86±0.36c | 0.38±0.00c | 275.18±0.49c | 7.19±0.04c | 2.58±0.16a | 0.003±0.00a | 0.75±0.03c | 1.82±0.13a |
|  | ASA1 | 35.00±0.20b | 0.50±0.00b | 295.00±2.81b | 10.50±0.00b | 3.38±0.00ab | 0.004±0.00ab | 0.79±0.01b | 2.58±0.02b |
|  | ASA2 | 38.00±0.22a | 0.58±0.02a | 310.00±2.84a | 12.00±0.43a | 2.69±0.09ab | 0.003±0.00ab | 0.94±0.01a | 1.75±0.07b |
| ^1^0h (*Normal condition*); 12h (*Cold stress*); 24h (*Cold stress*). ^2^ASA0, Acetylsalicylic acid (*0mM*); ASA1, Acetylsalicylic acid (*1mM*); ASA2, Acetylsalicylic acid (*2mM*). Pn, Photosynthesis rate; Gs, Stomatal conductance; Ci, CO_2_ assimilation; Tr, Transpiration rate; Chl, Total chlorophyll; Cx.c, Total carotenoids; Chla, Chlorophyll a; Chlb, Chlorophyll b. Data are mean ± standard deviation (SD). Correlation is two-tailed Student's t-tests were performed. P-values < 0.05 were considered statistically significant for both t-tests and ANOVA, respectively. | | | | | | | | | |

**Table S1b |** Impact of acetylsalicylic acid (ASA) on biochemical and antioxidant enzyme activity in common bean exposed to cold stress

| Treatment^1^ | Doses^2^ | EC | MDA | SP | SOD | POD | CAT | APX |
| --- | --- | --- | --- | --- | --- | --- | --- | --- |
|  |  | (*%*) | (*µmol g^-1^FW*) | (*gm. g^-1^*) | (*µg^-1^min^-1^FW*) | (*µg^-1^min^-1^*) | (*µg^-1^min^-1^*) | (*µg^-1^min^-1^*) |
| 0h | ASA0 | 90.79±0.6a | 0.02±0.00a | 123.11±0.75a | 546.88±27.52a | 24.38±2.2b | 82.28±5.86c | 140.72±2.78a |
|  | ASA1 | 88.94±2.08a | 0.02±0.00a | 36.71±1.85a | 433.71±2.03c | 44.83±1.13b | 130.25±5.75b | 146.42±2.4a |
|  | ASA2 | 55.17±1.64b | 0.01±0.00b | 42.99±0.65a | 834.17±2.30b | 70.34±3.42a | 169.8±3.47a | 131.88±3.83a |
| 12h | ASA0 | 90.32±1.32a | 0.02±0.00a | 117.79±1.19b | 318.69±1.95c | 28.6±1.68c | 98.76±1.74b | 122.6±1.12a |
|  | ASA1 | 89.74±1.29a | 0.02±0.00b | 42.06±2.17a | 678.43±3.99a | 60.2±0.77b | 153.81±1.68a | 83.68±3.77b |
|  | ASA2 | 96.39±1.46a | 0.01±0.00c | 38.74±1.32c | 417.35±1.88b | 73.63±2.75a | 215.99±2.61c | 77.14±1.91a |
| 24h | ASA0 | 88.4±2.55bc | 0.02±0.00c | 116.75±2.44a | 438.59±3.11a | 38.56±0.80a | 189.33±3.84c | 123.37±2.39b |
|  | ASA1 | 92.21±0.45a | 0.01±0.00a | 33.23±1.03a | 521.17±1.84c | 63.7±1.27a | 96.44±2.72a | 159.25±2.01c |
|  | ASA2 | 73.82±2.99b | 0.01±0.00b | 39.16±0.87b | 662.27±1.07b | 72.31±1.37a | 184.11±1.63b | 162.74±1.28a |
| ^1^0h (Normal condition); 12h (Cold stress); 24h (Cold stress). 2ASA0, Acetylsalicylic acid (0mM); ASA1, Acetylsalicylic acid (1mM); ASA2, Acetylsalicylic acid (2mM). EC, Electrical conductance; MDA, Malondialdehyde content; SP, Total soluble protein; SOD, Superoxide dismutase activity; POD, Peroxidase activity; CAT, Catalase activity; APX, Ascorbate peroxidase activity. Data are mean ± standard deviation (SD). Correlation is two-tailed Student's t-tests were performed. P-values < 0.05 were considered statistically significant for both t-tests and ANOVA, respectively. | | | | | | | | |

**Table S2 | Primer pairs used for RT-qPCR validation of 14 cold stress-responsive genes in common beans**

| Gene ID | Gene name | Forward primer 5'- 3' | Revers primer 5'- 3' |
| --- | --- | --- | --- |
| Phvul.011G071300 | *APX1* | AGGCGGAGTTCCCTATCTTG | GACCCCTTGGTTGCATCAG |
| Phvul.009G126500 | *APXT* | TCGCATTTCGCACCTGTT | GGACCTGAGAAGCTCCTTGATA |
| Phvul.009G197600 | *GLCAT14B* | GCAGGTGGTTCAGTCAAGCG | GGCATTCCAATGTCAGGTGT |
| Phvul.004G092694 | *hcb1* | GTGGTTCAAGGCTGGGTCTC | AATGCGATAACCCTCCACTG |
| Phvul.003G212400 | *LPCAT1* | TTGGGACCATTATTCCTGTTG | AAGCTGGAGACCCGTTCTACTA |
| Phvul.004G163900 | *NYC1* | GTGGGGATGCCAAGGACAA | ATCATAGTCACAATATACCT |
| Phvul.002G223900 | *POD1* | ACGGCTGAGTCGTCGGAGTT | TGGCAAGCGGAAGATAGTGT |
| Phvul.005G083700 | *POR* | CACTGTTCGGTCTTTCATTGTC | CCTCAGTGTTTTCTTGCCTTCT |
| Phvul.009G125100 | *PsbS* | CCAAGGTTACAAAGTCAAAGCC | CCAACAGTGATGCCGCAAA |
| Phvul.004G073400 | *RbcS1* | TGGCTTCTTCAATGATCTCCTC | CACTCTTCCACCGTTGTTCG |
| Phvul.011G086300 | *SODC* | AACTGGAAATGCTGGTGGAA | GGGATAACCCAAAGGGAAAA |
| Phvul.003G243800 | *ZE* | ATGCTGAGTTGGGTCTTAGGTG | GCTCAAGGGCTTCATCGTCT |
| Phvul.008G161000 | *CAO* | TCAGTCATACCATCCCCACTTG | TTTCCCTCTGAAGATAACCCAT |
| Phvul.001G132200 | *CHLH* | AAATCTGCCAGCCCTCATC | TGCATCACTCATCCCCACC |
| *β-*actin | Reference gene | TGCATACGTTGGTGATGAGG | AGCCTTGGGGTTAAGAGGAG |
